# Supplementary material for: Contribution of women’s preference to the overuse of caesarean sections: A propensity score matching analysis based on a multi-country cross-sectional survey, as part of the QUALI-DEC project
Source: PLoS One. 2025 Dec 18;20(12):e0339007. doi: 10.1371/journal.pone.0339007 (PMC12714225; doi:10.1371/journal.pone.0339007)
Supplement: S2 Table — (DOCX) [file pone.0339007.s002.docx]

Supplementary Table S2: Institutional variables of the hospitals where the study women delivered (QUALI-DEC)

| **Institutional variables*** | **Argentine (N=8)** | **Burkina Faso (N=8)** | **Thailand (N=8)** | **Viet Nam (N=8)** | **Total of women (N=2092)** |
| --- | --- | --- | --- | --- | --- |
| **Referral level, n (%)** |  |  |  |  |  |
| Primary – Secondary (ref) | 2 (25.0) | 6 (75.0) | 1 (12.5) | 7 (87.5) | 1119 (53.5) |
| Tertiary | 6 (75.0) | 2 (25.0) | 7 (87.5) | 1 (12.5) | 973 (46.5) |
| **Any private practice in the maternity unit, n (%)** |  |  |  |  |  |
| No (ref) | 7 (87.5) | 2 (25.0) | 2 (25.0) | 2 (25.0) | 971 (46.4) |
| Yes (private ward or facility) | 1 (12.5) | 6 (75.0) | 6 (75.0) | 6 (75.0) | 1121 (53.6) |
| **Teaching facility, n (%)** |  |  |  |  |  |
| No (ref) | 0 (0.0) | 5 (62.5) | 0 (0.0) | 2 (25.0) | 468 (22.4) |
| Yes | 8 (100.0) | 3 (37.5) | 8 (100.0) | 6 (75.0) | 1624 (77.6) |
| **Presence of a functioning US machine in delivery ward, n (%)** |  |  |  |  |  |
| No (ref) | 4 (50.0) | 4 (50.0) | 0 (100.0) | 1 (12.5) | 513 (24.5) |
| Yes | 4 (50.0) | 4 (50.0) | 8 (100.0) | 7 (87.5) | 1579 (75.5) |
| **Permanence of an anaesthetist, n (%)** |  |  |  |  |  |
| No (ref) | 7 (87.5) | 7 (87.5) | 2 (25.0) | 4 (50.0) | 1151 (55.0) |
| Yes | 1 (12.5) | 1 (12.5) | 6 (75.0) | 4 (50.0) | 941 (45.0) |
| **Any individual delivery room, n (%)** |  |  |  |  |  |
| No (ref) | 6 (75.0) | 8 (100.0) | 7 (87.5) | 6 (75.0) | 1564 (74.8) |
| Yes | 2 (25.0) | 0 (0.0) | 1 (12.5) | 2 (25.0) | 528 (25.2) |
| **Number of births per one midwife/nurse per day, median (Q1-Q3)** | 0.7 (0.5 – 0.8) | 1.4 (1.1 – 1.7) | 0.9 (0.8 – 1.0) | 1.2 (0.5 – 1.6) | 1.0 (0.8 – 1.4) |
| **Number of births per one obstetrician per day, median (Q1-Q3)** | 0.7 (0.6 – 0.8) | 5.1 (4.0 – 6.9) | 4.5 (3.5 – 6.6) | 2.9 (2.1 – 4.0) | 3.7 (2.0 – 4.9) |
| **Number of births per admission bed per day, median (Q1-Q3)** | 0.8 (0.7 – 1.3) | 2.3 (1.7 – 3.1) | 0.8 (0.7 – 0.9) | 1.8 (0.9 – 2.7) | 1.1 (0.8 – 2.1) |
| **Number of births per delivery bed per day, median (Q1-Q3)** | 1.0 (0.9 – 1.8) | 2.3 (1.7 – 3.1) | 2.6 (1.4 – 3.1) | 2.1 (1.8 – 2.9) | 2.1 (1.4 – 3.0) |

*Characteristics of the hospital where the woman delivered

US: Ultrasound
