# Supplementary figures and images for: Contribution of women’s preference to the overuse of caesarean sections: A propensity score matching analysis based on a multi-country cross-sectional survey, as part of the QUALI-DEC project
Source: PLoS One. 2025 Dec 18;20(12):e0339007. doi: 10.1371/journal.pone.0339007 (PMC12714225; doi:10.1371/journal.pone.0339007)

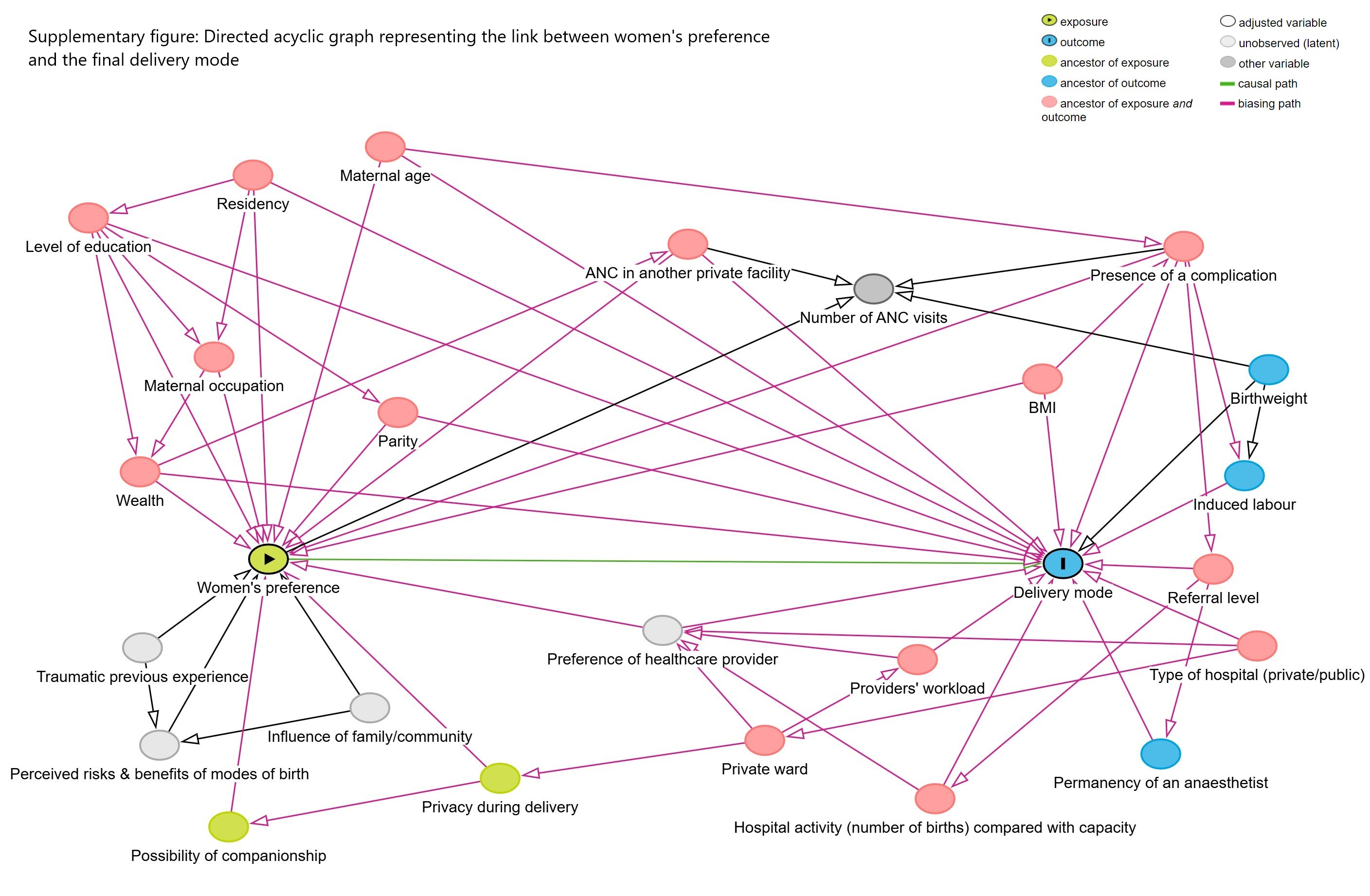

Supplement: S3 Fig — (TIFF) [file pone.0339007.s003.tiff]
