# Supplementary material for: Contribution of women’s preference to the overuse of caesarean sections: A propensity score matching analysis based on a multi-country cross-sectional survey, as part of the QUALI-DEC project
Source: PLoS One. 2025 Dec 18;20(12):e0339007. doi: 10.1371/journal.pone.0339007 (PMC12714225; doi:10.1371/journal.pone.0339007)
Supplement: Attachment — Submitted filename: Review comments.docx [file pone.0339007.s004.docx]

Review comments

First I would like to say thank you the editors for invited me to review this article entitled **“Contribution of women's preference to the overuse of caesarean section: a propensity score matching analysis based on a multi-country cross-sectional survey, as part of the QUALI-DEC project.” With** Manuscript Number: PONE-D-25-15077

1. Generally it is well written paper and targeted its objectives.
2. Used advanced English language
3. However I have some question section by section

**Abstract**

1. Your title and your objectives in abstract section are not similar? Why? Line#33
2. You wrote CS was high due to maternal request, if it was high but your argument was lack of evidence it was paradox; why you want to conduct this title? Line #32
3. The sample size was not clear because it seems goes to numbers of stages; in addition you took 71 participants in each hospital. If so 71*32=2272 but your sample size was 1827. Why? It needs deep explanation.

**Introduction**

4. The argument you stated is convincing. Why you want to conduct? How we know you were free from the projects influence that may have impact on the result of this paper?

**Methods and materials**

5. How you selected the study areas from other LMICs? It did not show the probability sampling technique. If so your generalization of the results of this paper may in questioned?
6. Your participant in each hospital was 71 for minimum of two-week. Then you stated that if you reached the total sample (71) before two-weeks you continued your data collection till two-weeks. Why? Line# 182.
7. This sentence is clear for me “For high-activity 186 hospitals, a randomization factor was applied each day to all women who had given birth the 187 previous day to obtain a random sample of 10 women, assuming that between 4 and 5 women 188 would refuse to participate or would not be eligible.” Line# 186-188.

**Result:** No need of revision.

**Discussion:** I think it was your limitation *“The multi-site and multi-country design is a strong point of this study, allowing the 420 results to be generalized to similar contexts.”* Because your sampling technique was not purely probable type. You used nonprobability (purposive) type.
