# Supplementary material for: Contribution of women’s preference to the overuse of caesarean sections: A propensity score matching analysis based on a multi-country cross-sectional survey, as part of the QUALI-DEC project
Source: PLoS One. 2025 Dec 18;20(12):e0339007. doi: 10.1371/journal.pone.0339007 (PMC12714225; doi:10.1371/journal.pone.0339007)
Supplement: Attachment — Submitted filename: Responses to Reviewers.docx [file pone.0339007.s006.docx]

| **Reviewer #1** |  |
| --- | --- |
| **1. Clarify Causal Inference:** While PSM reduces confounding, the manuscript overstates causal claims (e.g., "the fraction of CS attributable to women’s preference"). Emphasize that the design is observational and that unmeasured confounders (e.g., clinician influence, financial incentives) may bias results. Consider using terms like "association" rather than "attributable." | Thank you for your comment.  We agree that the observational design does not allow us to claim a causal link. We have therefore used the term “associated” (e.g., “fraction of CS associated with women's preference”) instead of “attributable,” as you recommended.  This limitation is emphasized in the following sentences in the discussion section:  *“However, we cannot definitely establish a causal relationship between women's preferences and their mode of birth, as the data are cross-sectional and do not account for the temporality of events. For this reason, we have not used the term “attributable fraction” but rather “associated fraction”. Therefore, the findings on associated fractions provide an indication of the contribution of women's preference to the use of CS and should be interpreted with caution.”*  Page 21-22, Lines 507-512 of the revised manuscript (without track changes) |
| **2. Address Recall Bias:** Women’s preferences were assessed postpartum, which risks recall bias and reverse causality (preference influenced by delivery outcome). Expand the discussion of how this might inflate the observed association and suggest future studies prospectively measure preferences during pregnancy. | Thank you for your comment.  We have expanded the discussion as recommended, with the following sentences:  “*Women may have been biased toward reporting a preferred mode of delivery consistent with the type of delivery they had just experienced, thereby inflating the observed association.* *We think that adjusting the measure on actual mode of delivery may control partially this recall bias. The more appropriate measure of the association between women's preferences and their mode of delivery would be a prospective study examining women’s preferences during pregnancy”*  Page 21, Lines 483-488 of the revised manuscript (without track changes) |
| **3. Contextualize Country-Specific Findings:** The high attributable fraction in Thailand (23%) and stark contrast in Vietnam (12%) warrant deeper exploration. Discuss potential cultural, institutional, or policy differences (e.g., Vietnam’s clinician influence on preferences) that might explain these variations. | Thank you for your comment.  Indeed, the proportion of CS associated with women's preferences varies across countries. The higher proportion of CS associated with women's preferences observed in Thailand is explained by the high prevalence of women's preference for CS in that country (19%) compared to Vietnam, where it is only 5%. This finding had already been mentioned in a previous Quali-DEC publication, in which we also observed that preference for CS was more pronounced among nulliparous women and was linked to the fear of pain, especially in Thailand. In this country, pain management is nearly non-existent, and nulliparous women account for more than half (51.6%) of our sample, which could explain the higher prevalence of women's preference for CS. Similarly, there is a strong belief in Thailand that the date of birth should be chosen at an auspicious time to ensure the destiny of the child and the family.  As the reviewer suggested, we have expanded the discussion section to incorporate these explanations. Please, see pages 18-19, Lines 425-435 of the revised manuscript (without track changes). We have also added references to the previous QUALI-DEC analysis. |
| **4. Strengthen the Discussion of Clinician/Systemic Factors:** While the conclusion mentions systemic factors, the manuscript underanalyzes how clinician preferences or hospital policies (e.g., financial incentives, fear of litigation) interact with women’s preferences. Cite qualitative findings from the QUALI-DEC project or existing literature to contextualize these dynamics. | Thank you for your comment.  We drew on previous qualitative studies to explain how clinician-related or systemic factors may explain the results. We revised the following section in the discussion:  *“In Burkina Faso, for example, general practitioners who lacked obstetric skills but worked in maternity wards due to a shortage of specialized staff were more likely to perform unnecessary CS. In Thailand, obstetricians describe vacuum or forceps extraction as requiring “a high level of expertise and skill” that they believe they do not have and consider CS to be the best option to avoid adverse events. In addition, CS is perceived by some doctors as a way to better manage their clinical and academic work and personal time, but also to ensure the baby’s safety, while vaginal delivery is perceived as more unpredictable in terms of neonatal outcomes. In Vietnam, healthcare professionals have reported experiencing considerable pressure, which explains why CS is often decided for fear of litigation or being targeted on social media, as CS is seen as a form of protection. This finding is also described in Thailand and Argentina. Financial incentives to perform CS rather than vaginal deliveries are regularly mentioned in the literature. This is probably the case in Thailand and Vietnam, where private practice within public hospitals, observed during the research but not yet documented, is widespread. Finally, certain organizational factors may contribute to the use of CS. A previous QUALI-DEC study showed that emergency intrapartum CS was significantly associated with a higher bed occupancy level, revealing clinicians' preference for performing CS in order to facilitate the organization of care and, in their opinion, ensure optimal quality of care. Furthermore, in Argentina, shortage of midwives in some hospitals or their limited role during labor and delivery, while obstetricians manage labor in most public hospitals, even for low-risk pregnancies, are described as factors that may contribute to excessive use of CS”*  Page 17-18, Lines 390-411 of the revised manuscript (without track changes) |
| **5. Data Availability:** The statement that data will be available by late 2024 is acceptable, but clarify how readers can access the data post-publication (e.g., Zenodo DOI, project website). | Thank you for your comment and your understanding.  The publication of Quali-Dec data is postponed to the end of 2025, as the data are still being analyzed (before-after evaluation). However, the data sharing plan is a requirement of the research protocol and will be applied as soon as possible.  We have added details on how readers can access the data. We have provided the web link that leads directly to the QUALI-DEC community on Zenodo, where the data will be published. |
| **6. Minor Revisions:** o Tables/Figures: Ensure consistency in terminology (e.g., "Viet Nam" vs. "Vietnam"). Simplify Table 1 for readability (e.g., merge redundant categories). o Abstract: Specify the timeframe of data collection (2020–2022) to contextualize the study period. o Introduction: Briefly define "low-risk" women using Robson classification criteria to aid non-specialist readers. | Thank you for your suggestions.   - We have ensured consistency in terminology when referring to Viet Nam. We have aligned ourselves with the terminology policy of the WHO and the United Nations, which use the two-word spelling “Viet Nam” in English. This terminology has been chosen to respect the official preference of the Vietnamese government. The two-word form is considered more respectful of the country's linguistic and cultural conventions. - Table 1 has been simplified as recommended (pages 13 and 14) - Abstract: We have added the data collection period (2020-2022) in the abstract. Page 2, Line 37 - Definition of low-risk women: “*Women belonging to groups 1 to 4 of the Robson classification are the target population for Quali-Dec [29].* *They are at lower risk of CS, as compared to groups 5, or groups 6 to 10*.” Page 4, Lines 108-109 |
|  |  |
| **Reviewer #2** |  |
| **Abstract** 1. Your title and your objectives in abstract section are not similar? Why? Line#33 | Thank you for your comment.  The objective has been harmonized between the abstract and the introduction. |
| 2. You wrote CS was high due to maternal request, if it was high but your argument was lack of evidence it was paradox; why you want to conduct this title? Line #32 | Thank you for your comment.  The increasing CS rates are often perceived to be associated with an increase in maternal demand for CS, as reported by many clinicians interviewed in studies available in scientific literature. However, this perception may not reflect reality, and current evidence supporting this claim is limited. Few hospital-based studies have investigated the link between maternal demand and CS rates, but these results are biased because maternal demand is usually poorly reported. For this reason, we thought that our study was important to fill the knowledge gap about the contribution of women’s preference and demand for CS in the increasing CS rates by measuring the real proportion of CS associated with women’s preference. |
| 3. The sample size was not clear because it seems goes to numbers of stages; in addition you took 71 participants in each hospital. If so 71*32=2272 but your sample size was 1827. Why? It needs deep explanation. | Thank you for your comment.  You are correct, the total sample was larger than the sample analyzed in this study. As shown in the flowchart, a total of 3,127 women were included in the survey. However, to reduce bias, we restricted our analysis to a sample of low-risk women defined as women with no history of CS, singleton pregnancy, cephalic presentation, and term delivery – 37 or more weeks of pregnancy), i.e. 1,827 women (also indicated in the flowchart). |
| **Introduction** 4. The argument you stated is not convincing. Why you want to conduct? How we know you were free from the projects influence that may have impact on the result of this paper? | Thank you for your comment.  Let me rephrase our argument. The commonly shared idea that rising CS rates are associated with increased maternal demand for CS was not supported by strong evidence. Few hospital-based studies have investigated the link between maternal demand and CS rates, but these results are biased because maternal demand is usually poorly reported. In participating countries of Quali-Dec project, CS on maternal request is not allowed. Measuring women's preference for CS rather than maternal demand is therefore more accurate in this context. By examining the proportion of CS associated with women’s preference, we thought we could obtain a good approximation of the proportion of CS performed at maternal request and confirm or refute the hypothesis that women's preference or demand for CS contributes to higher CS rates.  All researchers analyzing the data have no conflict of interest and are completely independent from both the funders and the management of the participating hospitals. This study was conducted as a secondary analysis following questions raised by the co-authors during the analysis of collected data. This study was neither commissioned nor influenced by external institutions or organizations. It stems from the researchers' desire to understand the context in which the QUALI-DEC research is being conducted for scientific purposes. |
| **Methods and materials** 5. How you selected the study areas from other LMICs? It did not show the probability sampling technique. If so your generalization of the results of this paper may in questioned? | The QUALI-DEC study is an implementation research. The participating countries were chosen because of the concern of local scientific and medical authorities about the significant increase in CS rates in many of their hospitals and its consequences. In addition, we found a clear determination among policymakers in these countries to revert this trend using evidence-based interventions. This project is also the result of a long collaboration between the researchers involved in this subject, who currently make up the QUALI-DEC consortium. The choice of these countries was therefore operational. A detailed description of the rational, methods and Theory of Change has been published in two scientific manuscripts reporting the protocols of the study (effectiveness and process evaluation protocols):  Cleeve et al., 2023, Global Health Action: <https://www.tandfonline.com/doi/full/10.1080/16549716.2023.2290636>  Dumont et al., 2020, Implementation Science: <https://implementationscience.biomedcentral.com/articles/10.1186/s13012-020-01029-4>  Indeed, the sampling technique was not probabilistic, which limits the generalizability of the results. We therefore qualified this point in the discussion:  “*The multisite and multicountry design is a strength of this study, as it could allow the results to be generalized to similar contexts, although it should be noted that our non-probabilistic sampling technique limits this generalizability*.”  Page 20, Lines 470-472 of the revised manuscript (without track changes) |
| 6. Your participant in each hospital was 71 for minimum of two-week. Then you stated that if you reached the total sample (71) before two-weeks you continued your data collection till two-weeks. Why? Line# 182. | That's right. Methodologically, it was necessary to have the same two-week collection period at each site to ensure a consistent procedure everywhere. When the sample size was not reached within two weeks, we prolonged the data collection period. This was necessary in 8 hospitals.  This approach has been used before in surveys: Bull World Health Organ. 2007 Nov 26;86(2):126–131. doi: [10.2471/BLT.06.039842](https://doi-org.ezproxy.u-paris.fr/10.2471/BLT.06.039842) |
| 7. This sentence is not clear for me “For high-activity hospitals, a randomization factor was applied each day to all women who had given birth the previous day to obtain a random sample of 10 women, assuming that between 4 and 5 women would refuse to participate or would not be eligible.” Line# 186-188. | Thank you for your comment.  We have changed this sentence for better clarity:  “*In hospitals with more than 10 deliveries per day, we randomly selected 10 women each day among all women who had given birth the previous day. Assuming that 4 to 5 women would refuse to participate or would not be eligible from this random sample, 5 to 6 women would be included in the survey each day, allowing the required number of subjects to be reached.”*  Page 8, Lines 204-209 |
| **Results**  No need of revision. | / |
| **Discussion**  I think it was your limitation “The multi-site and multi-country design is a strong point of this study, allowing the results to be generalized to similar contexts.” Because your sampling technique was not purely probable type. You used nonprobability (purposive) type. | Thank you for your comment.  We have nuanced this point and mentioned the limitation of the sampling technique as explained in Page 20, Lines 470-472 of the revised manuscript (without track changes) |
