# Supplementary material for: Contribution of women’s preference to the overuse of caesarean sections: A propensity score matching analysis based on a multi-country cross-sectional survey, as part of the QUALI-DEC project
Source: PLoS One. 2025 Dec 18;20(12):e0339007. doi: 10.1371/journal.pone.0339007 (PMC12714225; doi:10.1371/journal.pone.0339007)
Supplement: Attachment — Submitted filename: Response to Reviewers_R2.docx [file pone.0339007.s007.docx]

| **Reviewer’s comments** | **Authors’ responses** | **Revisions in the manuscript** |
| --- | --- | --- |
| **Reviewer #1** |  |  |
| My first comments were thoroughly addressed and carefully considered, leaving me genuinely satisfied with the response. |  |  |
| **Reviewer #2** |  |  |
| All my comments were properly addressed. Therefore, I have no additional comments and questions to be addressed again. |  |  |
| **Reviewer #3** |  |  |
| In your abstract section, abbreviation is not recommended, so omit it | We agree with this suggestion. We have omitted abbreviations in the abstract section. | Revised in the abstract section  *Page 2* |
| In introduction section, if so what interventions were done to decrease unnecessary C/S worldwide, in low and middle income countries specifically in your study area? | Interventions aimed at reducing the number of unnecessary C/S target pregnant women, healthcare professionals, and healthcare systems. For women, they mainly consist of educational activities, childbirth preparation workshops, and ongoing support during labor. For professionals, the most effective strategies include implementing evidence-based guidelines and clinical audits, while financial or regulatory measures are not supported by strong evidence. The most promising strategies must combine several interventions involving all stakeholders, addressing non-clinical determinants, and adapted to local contexts in LMICs. | Added in the introduction section  *Page 5, lines 111-118* |
| Line 60 how increasing rate of C/S affect countries? in which context? | Excessive use of C/S has been associated with increased rates of maternal and neonatal complications in LMICs. Unnecessary C/S also result in additional financial costs for health systems and individuals and constitute an obstacle to universal health coverage. Within the same country, there are inequalities whereby women with financial resources have access to C/S, sometimes for non-medical reasons, while women without resources do not have access to C/S even though this procedure can be life-saving. | Explanation provided in the introduction section  *Page 2, lines 68-73* *of the revised manuscript with track changes* |
| Line 87 odds of previous studies is not necessary | We agree with this suggestion. We have rephrased this sentence without mentioning the odds ratio. | Revised in the introduction section  *Page 4, lines 94-96* *of the revised manuscript with track changes* |
| Why women's who gave stillbirth or neonatal death before discharge excluded form your study? | Women who had given birth to a stillborn child or a child who died during the neonatal period were excluded for ethical reasons. In our study, women had to be interviewed after giving birth. Out of respect for the women's grief and for ethical reasons, we did not want to ask women who had lost their newborns to take the time to answer a questionnaire.  As the number of women excluded for these reasons was small, the risk of bias was considered low. | Clarification added in the method section  *Page 7, lines 181-182 of the revised manuscript with track changes* |
| During baseline survey data collection period was two weeks in each hospital with the required number of (n=71) for two weeks in each hospital, during this time if the required number is reached before two weeks duration, data collection was continued until the end of predefined period. | We apologize; we are not sure we understand your comment or question.  That is exactly what we did.  The data collection period for the baseline survey was two weeks, by default, in each hospital, to reach the required number of participants (n = 71). If the required number was reached before the end of the two weeks, data collection continued until the end of the predefined period. If the required number was not reached before the end of the two weeks, data collection continued until that number was reached. | Explanation provided in the method section  *Pages 8 & 9, lines 215-218* *of the revised manuscript with track changes* |
| If the tools were prepared by reviewing previous literatures, how did you check the validity? | The questionnaire was developed based on a literature review and content validation by all the experts comprising the QUALI-DEC research consortium. The questionnaire was then discussed and adapted with country investigators and local data collectors. Additionally, it was pre-tested among a sample of women before starting data collection. A few additional modifications were necessary to adapt it to the context and linguistic challenges, in order to ensure its validity. |  |
