# Supplementary material for: Contribution of women’s preference to the overuse of caesarean sections: A propensity score matching analysis based on a multi-country cross-sectional survey, as part of the QUALI-DEC project
Source: PLoS One. 2025 Dec 18;20(12):e0339007. doi: 10.1371/journal.pone.0339007 (PMC12714225; doi:10.1371/journal.pone.0339007)
Supplement: Attachment — Submitted filename: Response to Reviewers_R3.docx [file pone.0339007.s008.docx]

| **Reviewer’s comments** | **Authors’ responses** | **Revisions in the manuscript** |
| --- | --- | --- |
| 1. If the authors have adequately addressed your comments raised in a previous round of review and you feel that this manuscript is now acceptable for publication?  Reviewer #1: All comments have been addressed  Reviewer #3: (No Response) |  |  |
| 2. Is the manuscript technically sound, and do the data support the conclusions?  Reviewer #1: Yes  Reviewer #3: (No Response) |  |  |
| 3. Has the statistical analysis been performed appropriately and rigorously?  Reviewer #1: Yes  Reviewer #3: (No Response) |  |  |
| 4. Have the authors made all data underlying the findings in their manuscript fully available?  Reviewer #1: Yes  Reviewer #3: (No Response) |  |  |
| 5. Is the manuscript presented in an intelligible fashion and written in standard English?  Reviewer #1: Yes  Reviewer #3: (No Response) |  |  |
| Reviewer #1: No major modifications are necessary. However, to further strengthen clarity and precision before final submission, consider:  Abstract (lines 29–53):  Suggest simplifying the phrasing in the final sentence to make the policy implication more direct (e.g., “...highlighting the need for multidimensional, context-specific strategies to reduce unnecessary caesarean sections.”) | Thank you for your comment.  We have simplified this sentence according to your recommendation. | Revised in the abstract section  *Page 2, lines 49-54 of the manuscript with track changes* |
| Statistical section (lines ~244–317):  Add a brief explanation of why the nearest-neighbour matching with replacement was chosen over caliper or kernel methods (for transparency and reproducibility). | Thank you for your comment.  We have added a brief explanation of why the nearest-neighbour matching with replacement was chosen. | Added in the statistical section  *Page 11, lines 298-302 of the manuscript with track changes* |
| Discussion:  The interpretation is comprehensive, but the discussion could briefly note the potential for unmeasured confounding, particularly provider attitudes or institutional norms not captured in the data. | Thank you for your comment.  We have added a brief note regarding potential unmeasured confounding factors in the section on limitations. | Added in the discussion section  *Page 22, lines 520-522 of the manuscript with track changes* |
| A short paragraph linking the study’s findings to WHO’s recommendations on CS reduction could strengthen its applied relevance. | Thank you for your comment.  We have added a short paragraph linking the study’s findings to WHO’s recommendations on CS reduction. | Added in the discussion section  *Page 21, lines 309-320 of the manuscript with track changes* |
| Formatting:  Ensure consistent spacing and punctuation (minor spacing issues appear intermittently in the Results tables and references). | Thank you for your comment.  We have checked the consistency of spacing and punctuation throughout the manuscript. |  |
| Verify all references are formatted per PLOS ONE style (check capitalization, italics, and DOI availability). | Thank you for your comment.  All references are formatted per PLOS ONE style. | Revised in the reference section |
